# Is ultrasound training sustainable? A systematic review of competency retention in healthcare trainees

**DOI:** 10.1111/medu.15751

**Published:** 2025-06-16

**Authors:** Liang‐Wei Wang, Cheng‐Heng Liu, Wen‐Yi Li, Wen‐Chu Chiang, Yen‐Lin Chiu, Matthew Huei‐Ming Ma, Huey‐Ling Chen, Chih‐Wei Yang

**Affiliations:** ^1^ Department of Medical Research and Education National Taiwan University Hospital, Yunlin Branch Yunlin Taiwan; ^2^ Department of Emergency Medicine National Taiwan University Hospital, Yunlin Branch Yunlin Taiwan; ^3^ Graduate Institute of Medical Education and Bioethics National Taiwan University College of Medicine Taipei Taiwan; ^4^ Department of Medical Education National Taiwan University Hospital Taipei Taiwan; ^5^ Department of Emergency Medicine National Taiwan University Hospital Taipei Taiwan; ^6^ Division of Nephrology, Department of Internal Medicine National Taiwan University Hospital Yunlin Branch Yunlin Taiwan; ^7^ College of Medicine National Taiwan University Taipei Taiwan

## Abstract

**Purpose:**

Despite Point‐of‐Care Ultrasound (PoCUS) emerging as an essential clinical skill, evidence regarding practitioners' knowledge retention and competence remains poorly synthesized. This systematic review sought to evaluate PoCUS competency retention patterns using the Indication, Acquisition, Interpretation, Medical decision‐making (I‐AIM) framework following various educational interventions and to identify factors influencing long‐term skill retention among healthcare professional trainees.

**Methods:**

The authors conducted a systematic review by searching PubMed, the Cochrane Library and Embase databases (1990–2024) for studies evaluating PoCUS educational interventions with objective competency measures and retention assessment. Data were analysed based on educational strategies, course duration and retention patterns across I‐AIM domains.

**Results:**

Thirty‐one studies met the inclusion criteria, comprising 1638 participants (38.6% medical students, 20.5% attending physicians). Most studies employed a single‐group pretest‐posttest design (54.8%) and demonstrated moderate‐to‐high methodological quality (Medical Education Research Study Quality Instrument median: 12.5, interquartile range [IQR]: 11.0 to 13.0). Within the I‐AIM framework analysis, all domains of competency retention demonstrated a decline over a 1–12‐month follow‐up period. The Acquisition domain showed the most significant decline with median percentage changes of −11.8% (IQR: −16.5% to −6.4%), followed by Interpretation, Medical decision‐making and Indication. Short‐course programs (≤4 h) demonstrated greater competency decline (median: ‐11.8%, IQR: −16.9% to −4.4%) compared to long‐course programs (median: ‐2.6%, IQR: −6.8% to 1.3%). Hands‐on practice with high‐fidelity simulation and clinical context integration were associated with superior retention outcomes across multiple domains.

**Conclusions:**

Ultrasound competence retention showed variable decay patterns across I‐AIM domains, with Acquisition skills showing the most pronounced deterioration, particularly following short‐course programs. Comprehensive training programs integrating high‐fidelity hands‐on practice and clinical context may enhance PoCUS retention for healthcare providers.

## Abstract 2

Evidence‐based tips for skill retention are offered through this systematic review of how training duration & methods affect PoCUS competency. #MedEd #POCUS

## 1 INTRODUCTION

Point‐of‐Care Ultrasound (PoCUS), pioneered by Rozycki et al in the early 1990s,^1^ has revolutionized modern clinical practice as “the new stethoscope” of 21st‐century medicine. As a bedside diagnostic modality employed by clinicians for real‐time assessment, PoCUS has demonstrated substantial utility in enhancing diagnostic accuracy, guiding clinical decision‐making processes and optimizing procedural safety.^2^, ^3^, ^4^, ^5^, ^6^, ^7^ Multiple medical specialties, including anaesthesia, orthopaedics, internal medicine and emergency medicine, have systematically integrated ultrasound training programs into their curricula.^8^, ^9^, ^10^, ^11^ Educational interventions have evolved to encompass diverse methodologies, including traditional didactic lectures,^12^, ^13^ blended learning models that incorporate multimedia‐based tools,^14^, ^15^ web‐based learning,^16^, ^17^, ^18^ simulation training,^8^, ^19^ and hands‐on practical sessions.^20^, ^21^ Nevertheless, long‐term retention of competence remains a critical concern in healthcare professional education, especially for resource‐intensive ultrasound training programs like PoCUS. Although medical educators recognize the potential need for regular refresher training similar to the 6–12‐month retraining programs implemented with adult advanced life support (ALS),^22^ limited evidence exists to comprehensively support and elaborate the competence retention of PoCUS.^23^

The Indication–Acquisition–Interpretation–Medical decision‐making (I‐AIM) framework was originally proposed as a mnemonic checklist for performing focused sonography, aiming to enhance knowledge retention by organizing the essential steps of the procedure into a simple, memorable structure. This stepwise framework guides practitioners through four key components: (1) determining appropriate clinical indications and medical necessity; (2) acquiring high‐quality images through optimal patient positioning, probe selection and technical adjustments; (3) systematically interpreting sonographic findings using pattern recognition and anatomical relationships; and (4) integrating these findings into clinical decision‐making within the broader clinical context.^24^ The I‐AIM framework has been applied across diverse clinical settings, including cardiac, pulmonary, abdominal, vascular and procedural interventions.^25^, ^26^, ^27^, ^28^ Furthermore, it has been directly implemented for assessing PoCUS competency, aligning with the principles of competency‐based medical education.^29^ Hence, the I‐AIM framework offers a practical and contextually relevant approach that supports learning, application and evaluation throughout PoCUS education.

In this systematic review, we aimed to examine the retention patterns of PoCUS competence based on the I‐AIM framework following various training curricula among healthcare professional trainees. Additionally, we sought to identify the educational intervention factors that influence long‐term retention of PoCUS competency.

## 2 METHODS

### 2.1 Study design and setting

We conducted this systematic review in accordance with the Preferred Reporting Items for Systematic Reviews and Meta‐Analyses (PRISMA) guidelines. We registered the review protocol in the International Prospective Register of Systematic Reviews (PROSPERO ID: CRD42025646291). We performed a comprehensive electronic literature search across three databases: PubMed (MEDLINE), Cochrane Library and Embase, covering studies published between January 1990 and December 2024. Our search strategy incorporated keywords such as “point‐of‐care ultrasound,” “skill retention,” and “medical education.” The complete search algorithm is detailed in Table 1. Two reviewers independently screened the titles and abstracts of all identified studies for eligibility. We reviewed full‐text articles deemed potentially relevant for inclusion, resolving any disagreements through discussion until consensus was achieved.

**TABLE 1:** Search strategies.

1. PICOTS framework for the research question	
Population	Healthcare providers (physicians, residents, medical students, nurses, nurse practitioner, paramedics, emergency medical technicians)
Intervention	Ultrasound training curricula include various educational methods, such as traditional lectures, flipped classrooms, team‐based learning and so on.
Comparison	No such notification
Outcomes	Knowledge and skill retention can be assessed through various objective assessment methods, including multiple‐choice examinations, Objective Structured Clinical Examinations (OSCE), standardized practical assessments, or quantitative performance metrics.
Study Design	Randomized controlled trials (RCTs) and non‐randomized studies (non‐randomized controlled trials, interrupted time series, controlled before‐and‐after studies, cohort studies and case series where *n* > 5) are eligible for inclusion.
Timeframe	From January 1990 to December 2024

2. Keywords	
Concept	Keyword
Ultrasound‐related terms	“Ultrasonography”, “Sonography”, “Point‐of‐care ultrasound”
AND	
Education‐related terms	“Education”, “Curriculum”, “Simulation”, “Training”
AND	
Population‐specific terms	“Education, Medical”, “Nurses”, “Paramedics”, “Emergency Medical Technicians”, “Students, Medical”, “Resident Education”, “Nurse Practitioner”
AND	
Outcome‐related terms	“Retention, Psychology”, “Memory”, “Interval”, “Skill Acquisition”, “Retention time”, “Skill retention”

3. Current search strategy:	
Pubmed	(“Sonography” OR “ultrasound” OR “Point‐of‐care ultrasound”) AND (“Education”[Mesh] OR “Curriculum”[Mesh] OR “Simulation”) AND (“Education, Medical”[Mesh] OR “Nurses”[Mesh] OR “Paramedics”[Mesh] OR “Emergency Medical Technicians”[Mesh] OR “Students, Medical”[Mesh] OR “Resident education” OR “Nurse practitioner”) AND (“Retention, Psychology”[Mesh] OR “Retention” OR “memory” OR “interval” OR “skill acquisition” OR “retention time” OR “Skill retention”). Filters (publication date): from 1990/01/01–2024/12/31
Cochrane Library	“Sonography” OR “ultrasound” OR “Point‐of‐care ultrasound” in Title Abstract Keyword AND “Education” OR “Curriculum” OR “Simulation” OR “Training” in Title Abstract Keyword AND “Education, Medical” OR “Nurses” OR “Paramedics” OR “Emergency Medical Technicians” OR “Students, Medical” OR “Resident education” OR “Nurse practitioner” in Title Abstract Keyword AND “retention time” OR “skill retention” OR “retention” OR “memory” OR “interval” OR “skill acquisition” in Title Abstract Keyword ‐ (Word variations have been searched) Limit with Cochrane Library publication date from Jan 1990 to Dec 2024, in Trials
Embase	#5. #1 AND #2 AND #3 AND #4 #4. ‘long term memory’ OR ‘retention’ OR ‘memory’ OR ‘interval’ OR ‘skill acquisition’ OR ‘retention time’ #3. ‘medical education’ OR ‘nurse’ OR ‘paramedical personnel’ OR ‘rescue personnel’ OR ‘medical student’ OR ‘resident education’ #2. ‘education’ OR ‘curriculum’ OR ‘simulation’ OR ‘training’ #1. ‘sonography’/exp OR ‘sonography’ OR ‘ultrasound’/exp OR ‘ultrasound’ OR ‘point‐of‐care ultrasound’/exp OR ‘point‐of‐care ultrasound’

### 2.2 Eligibility criteria

We included studies published in English if they met the following criteria: (1) participants were healthcare professional trainees, including physicians, residents, medical students, nurses, nurse practitioners, paramedics and emergency medical technicians; (2) the research evaluated PoCUS training curricula with both immediate (≤3 weeks) and delayed (>3 weeks) post‐educational assessments; and (3) the study reported objective assessment methods for knowledge and skills, such as multiple‐choice examinations, Objective Structured Clinical Examinations, standardized practical assessments, or quantitative performance metrics. We excluded studies if they were limited to subjective outcomes (e.g., self‐assessment, confidence measures, or self‐reported usage frequency). This exclusion criterion was applied because our study focused on the long‐term retention of objective performance, which can be more reliably measured and compared using standardized tools. Conversely, subjective outcomes are inherently variable, less reliable and not well‐suited for evaluating sustained competence. Studies that lacked quantifiable data for post‐educational or follow‐up assessments, as well as reviews, abstracts, conference papers and case reports, were also excluded.

### 2.3 Quality assessment

Two independent reviewers evaluated the methodological quality of the included studies using the Medical Education Research Study Quality Instrument (MERSQI), a validated assessment tool. We resolved any discrepancies through discussion until consensus was achieved. The MERSQI score assesses overall research quality across six domains: study design, sampling (including the number of institutions and response rate), type of data, validity evidence for evaluation instrument scores, data analysis (incorporating sophistication and appropriateness) and outcome measures. The total MERSQI score ranges from 5 to 18, with higher scores indicating a more rigorous methodology.^30^

### 2.4 Data extraction and statistical analysis

We performed data extraction using a standardized form capturing study characteristics (study design, first author, publication year), participant demographics (professional role and sample size) and detailed educational intervention parameters, such as pedagogical methodologies and intervention duration. We documented assessment metrics, including follow‐up intervals (in months), assessment objectives and evaluation methodologies and classified evaluation methods according to the I‐AIM framework, which encompasses four domains: indication, acquisition, interpretation and medical decision‐making.^24^ Educational outcomes were categorized using Kirkpatrick's training evaluation model at Levels 2 (learning) ‐ knowledge and skill acquisition; 3 (behaviour) ‐ clinical practice implementation; and 4 (results) ‐ organizational and patient outcomes; Level 1 (reaction) was excluded.^31^ We quantified performance change as the percentage difference between follow‐up and immediate post‐intervention assessment scores, normalized to the immediate post‐intervention score. Given the anticipated heterogeneity in study designs and outcome measures, we employed descriptive statistics, primarily median and interquartile ranges (IQRs), to characterize the distribution of performance changes across various assessment domains and temporal time points.

## 3 RESULTS

### 3.1 Characteristics of study participants

**FIGURE 1:**
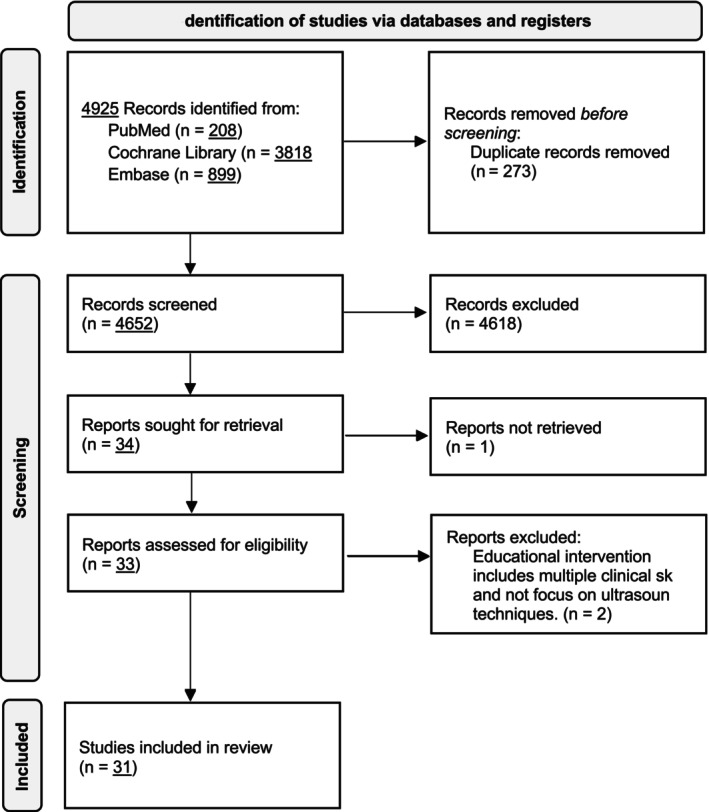
PRISMA flow diagram.

The systematic search identified 4925 citations across three databases: PubMed (*n* = 208), Cochrane Library (*n* = 3818) and Embase (*n* = 899). After removing duplicates, screening abstracts and reviewing full texts, 33 studies were retrieved. Two studies were excluded owing to their focus on general clinical skills assessment rather than specific ultrasound performance evaluation, resulting in 31 studies that met the inclusion criteria (Figure 1).

Most studies employed a single‐group pretest‐posttest design (54.8%, *n* = 17), followed by randomized controlled trials (38.7%, *n* = 12) and two‐group nonrandomized experimental studies (6.5%, *n* = 2). According to Kirkpatrick's training evaluation model, all studies were Level 2, with no higher‐level evaluations identified. The methodological quality was moderate‐to‐high, as evidenced by MERSQI scores of 9.0–14.0 (median: 12.5, IQR: 11.0–13.0). Lower scores were predominantly due to limitations in implementing randomized controlled designs and insufficient validity evidence for assessment instruments. Table 2 presents the detailed quality assessment results.

**TABLE 2:** MERSQI score.

No	First author (year published)	Total score	Study design	Sampling		Type of data	Validity evidence for evaluation instrument scores	Data analysis		Outcome
				Institutions	Response rate			Sophistication	Appropriate	
1	Toru Yamada (2023)	**13.0**	1.5	1.5	1.5	3	1	2	1	1.5
2	Christina Weaver (2023)	**11.0**	1.5	0.5	1.5	3	0	2	1	1.5
3	Mariam Haji‐Hassan (2022)	**10.5**	2	0.5	0.5	3	0	2	1	1.5
4	Jessica Szydlowski Pitman (2022)	**11.5**	1.5	0.5	1	3	1	2	1	1.5
5	Satoshi Jujo (2022)	**11.0**	1.5	0.5	0.5	3	1	2	1	1.5
6	Amy Kule (2022)	**13.5**	3	0.5	1.5	3	1	2	1	1.5
7	Satoshi Jujo (2021)	**14.0**	1.5	0.5	1.5	3	3	2	1	1.5
8	Christopher K. Schott (2021)	**12.5**	1.5	1.5	1	3	1	2	1	1.5
9	Yang Zhao (2021)	**12.5**	3	0.5	1.5	3	0	2	1	1.5
10	Gustavo Bittencourt Camilo (2021)	**12.5**	3	0.5	1.5	3	0	2	1	1.5
11	Swapna Thampi (2020)	**13.5**	3	0.5	1.5	3	1	2	1	1.5
12	Aun Woon Soon (2020)	**13.5**	3	0.5	1.5	3	1	2	1	1.5
13	Charles A. Rappaport (2019)	**11.0**	1.5	0.5	1.5	3	0	2	1	1.5
14	Samuel Larrivée (2019)	**11.0**	1.5	0.5	1.5	3	0	2	1	1.5
15	Carlos Augusto M. Menegozzo (2019)	**10.0**	1.5	0.5	0.5	3	0	2	1	1.5
16	Sebastian Ochoa (2019)	**12.5**	3	0.5	1.5	3	0	2	1	1.5
17	Rebekah J. McCurdy (2019)	**13.5**	3	1.5	1.5	3	0	2	1	1.5
18	Amanda M. Kleiman (2018)	**12.5**	3	0.5	1.5	3	0	2	1	1.5
19	Glenio B. Mizubuti (2018)	**11.0**	1.5	0.5	1.5	3	0	2	1	1.5
20	Ryan Breslin (2018)	**9.0**	1.5	0.5	1.5	3	0	1	0	1.5
21	Amanda M. Kleiman (2017)	**12.5**	3	0.5	1.5	3	0	2	1	1.5
22	Eun Jung Park (2017)	**12.5**	3	0.5	1.5	3	0	2	1	1.5
23	Ryo Yamamoto (2017)	**12.0**	1.5	0.5	1.5	3	1	2	1	1.5
24	Ankeet Deepak Udani (2016)	**13.5**	3	0.5	1.5	3	1	2	1	1.5
25	James A. Town (2016)	**12.0**	1.5	0.5	1.5	3	1	2	1	1.5
26	Diana J. Kelm (2015)	**12.5**	2	0.5	1.5	3	1	2	1	1.5
27	Thomas Edrich (2014)	**13.5**	3	0.5	1.5	3	1	2	1	1.5
28	Scott M. Thomas (2013)	**13.0**	1.5	0.5	1.5	3	2	2	1	1.5
29	Matthew Lyon (2012)	**10**	1.5	0.5	1.5	3	0	1	1	1.5
30	Oksana H. Baltarowich (2009)	**12.0**	1.5	1.5	1.5	3	0	2	1	1.5
31	Barry B. Goldberg (2001)	**11.0**	1.5	1.5	0.5	3	0	2	1	1.5

The studies included a total of 1638 participants, with the following distribution: medical students (38.6%), attending physicians (20.5%), resident physicians (17.7%), nurse practitioners (2.0%), registered nurses (1.0%) and others (20.2%). Overall, 43 courses with various educational interventions were analysed. Most PoCUS curricula (69.8%, *n* = 30) employed in‐person lecture‐based teaching. A substantial proportion (76.7%, *n* = 33) of the analysed curricula incorporated hands‐on practical training components. The median training duration was 2 h (range: 18 min–3 months), with 81.4% (*n* = 35) of curricula lasting <4 h. Of all PoCUS applications evaluated, cardiac ultrasonography was the most prevalent focus area (*n* = 14). Follow‐up assessments were conducted at various post‐intervention intervals, with the most common being 1 and 6 months (range: 1–12 months) (Table 3).

**TABLE 3:** Summary of 31 articles included in review.

No	First author (year published)	Subjects (*N*)	Study design	Educational methods	Hands‐on practice	Educational duration	Follow‐up time point (months)	Educational objectives and evaluation	Medical decision‐making (Δ%)	Indication (Δ%)	Interpretations (Δ%)	Acquisition (Δ%)	Kirkpatrick model level	MERSQI score
**1**	Toru Yamada (2023)	Nurse practitioners (33)	Single‐group pretest and posttest design	In‐person lectures	O	2 days	4.0	Cardiac, Lung, Abdominal, Vascular (lower extremity DVT)	X	X	−1.1%	−1.3%	**2**	**13.0**
**2**	Christina Weaver (2023)	First‐year osteopathic medical students (101)	Single‐group pretest and posttest design	In‐person lectures with Online lecture	O	1.3 hours	2.3	Musculoskeletal	−22.5%	X	−29.9%	X	**2**	**11.0**
**3**	Mariam Haji‐Hassan (2022)	First−/second‐year medical students (141)	Two‐group nonrandomized cohort design	Online lecture	X	8 hours	6.0	Cardiac	X	X	−37.5%	X	**2**	**10.5**
**4**	Jessica Szydlowski Pitman (2022)	Registered nurse anaesthetists (17)	Single‐group pretest and posttest design	Online lecture	O	4 hours	3.0	Ultrasound‐Guided vascular access (peripheral intravenous catheters, arterial catheters, central venous catheters)	−17.0%			−11.1%	**2**	**11.5**
**5**	Satoshi Jujo (2022)	First−/second‐year medical students (54)	Single‐group pretest and posttest design	In‐person lectures with Online lecture	O	2 hours	2.0	Cardiac	X	X	−9.2%	−39.2%	**2**	**11.0**
**6**	Amy Kule (2022)	First−/second‐year medical students (48)	Randomized controlled trial design	Group 1: Video learning;Group 2: Video learning, 4 additional mastery level simulated performances;Group 3: Video learning, 8 additional mastery level simulated performances;	O	1 hours	6.0	Ultrasound‐Guided vascular access (peripheral intravenous catheters)	X	X	X	Group 1: −8%; Group 2: 64%; Group 3: 36.5%	**2**	**13.5**
**7**	Satoshi Jujo (2021)	Second‐year medical students (6)	Single‐group pretest and posttest design	In‐person lectures with Online lecture	O	1.5 hours	2.0	Cardiac	X	X	−11.1%	−20.9%	**2**	**14.0**
**8**	Christopher K. Schott (2021)	Practicing physicians (127)	Single‐group pretest and posttest design	In‐person lectures	O	2.5 days	8.0	Cardiac	−3.7%		−9.1%		**2**	**12.5**
								Lung			−2.6%			
								Abdomen			−9.0%			
								Ultrasound‐guided peripheral intravenous catheter insertion			−2.6%			
**9**	Yang Zhao (2021)	Fourth‐year undergraduate medical students (120)	Randomized controlled trial design	Group 1: In‐person lectures with hands‐on practice on simulator; Group 2: In‐person lectures with video learning	O/X	2.5 hours	1.0	Cardiac (Transesophageal echocardiography)	X	X	Group 1: −17.3%;Group 2: −8.2%	X	**2**	**12.5**
**10**	Gustavo Bittencourt Camilo (2021)	Medical students, interns, paediatricians (42)	Randomized controlled trial design	Group 1: In‐person lectures with hands‐on practice; Group 2: In‐person lectures only	O/X	4 hours	6.0	Transfontanellar ultrasound	Group 1: −3.4%;Group 2: −13.3%			X	**2**	**12.5**
**11**	Swapna Thampi (2020)	Anesthesiologists (43)	Randomized controlled trial design	Group 1: In‐person lectures & 3D model presentation, followed by hands‐on practice on simulator; Group 2: Hands‐on practice on simulator, followed by in‐person lectures & 3D model presentation	O	3 hours	1.0	Cardiac (Transesophageal echocardiography)	X	Group 1: −11.1%; Group 2: −2.6%		Group 1: −11.8%; Group 2: −0.5%	**2**	**13.5**
**12**	Aun Woon Soon (2020)	Paediatric physicians, fellows, advanced practice providers (148)	Randomized controlled trial design	Group 1: In‐person lectures; Group 2: Online lecture	O	0.7 hours	2.0	Lung (pneumothorax, pleural effusion)	X	X	X	Group 1: −14.7%; Group 2: −12.1%	**2**	**13.5**
**13**	Charles A. Rappaport (2019)	First‐year medical students (24)	Single‐group pretest and posttest design	In‐person lectures	O	2 hours	1.0	Cardiac	X	X	−8.9%	−12.9%	**2**	**11.0**
							2.0				−2.7%	−25.8%		
							1.0	Lung			−29.8%	−11.8%		
							2.0				−45.7%	−11.8%		
							1.0	Vascular (IVC, IJV)			−4.2%	−16.7%		
							2.0				−2.1%	−16.7%		
**14**	Samuel Larrivée (2019)	Orthopaedic surgery residents (10)	Single‐group pretest and posttest design	Online lecture	O	6 hours	6.0	Musculoskeletal	−10.0%			−15.8%	**2**	**11.0**
**15**	Carlos Augusto M. Menegozzo (2019)	1st‐ to 4th‐ year medical students (37)	Single‐group pretest and posttest design	In‐person lectures	O	3.3 hours	3.0	E‐FAST	X	−5.1%	−35.3%	X	**2**	**10.0**
**16**	Sebastian Ochoa (2019)	First‐year medical students (20)	Randomized controlled trial design	Group 1: In‐person lectures; Group 2: In‐person lectures on 3D heart model	O	2 hours	3.0	Cardiac	X	X	Group 1: −24.3%; Group 2: −35.6%	X	**2**	**12.5**
**17**	Rebekah J. McCurdy (2019)	OB/GYN residents (57)	Randomized controlled trial design	Educational DVD lectures	X	1.5 hours	8.0	Transvaginal	−4.5%			X	**2**	**13.5**
**18**	Amanda M. Kleiman (2018)	Second‐year medical students (40)	Randomized controlled trial design	Group 1: Generative retrieval practice with video learning; Group 2: Video learning only	X	1 hours	1.0	Cardiac	X	X	Group 1: −5.9%; Group 2: −1.7%	X	**2**	**12.5**
							7.5				Group 1: 0%; Group 2: −0.6%			
**19**	Glenio B. Mizubuti (2018)	Senior anaesthesia residents (7)	Single‐group pretest and posttest design	In‐person lectures with Online lecture	O	1 month	6.0	Cardiac	X	−2.5%	−6.5%	X	**2**	**11.0**
**20**	Ryan Breslin (2018)	Foundation Year 1 doctors (20)	Single‐group pretest and posttest design	In‐person lectures	O	2 hours	1.0	Ultrasound‐Guided vascular access (peripheral intravenous catheters)	X	X	X	−5.9%	**2**	**9.0**
							3.0					−11.8%		
**21**	Amanda M. Kleiman (2017)	Fourth‐year medical students & anesthesiology residents (30)	Randomized controlled trial design	Group 1: Generative retrieval practice with video learning; Group 2: Video learning only	X	1.25 hours	1.0	Cardiac (Transesophageal echocardiography)	X	X	Group 1: −7.8%; Group 2: −12.2%	X	**2**	**12.5**
**22**	Eun Jung Park (2017)	Emergency medical technician (EMT) student (77)	Randomized controlled trial design	Group 1: In‐person lectures with video learning of real B‐lines; Group 2: In‐person lectures with video learning of simulated B‐lines	X	0.3 hours	2.0	Lung(B‐line)	X	X	Group 1: −14%;Group 2: −15.2%	X	**2**	**12.5**
**23**	Ryo Yamamoto (2017)	2−/3‐year medical students & PGY‐1 residents (31)	Single‐group pretest and posttest design	In‐person lectures	O	2 hours	1.0	Cardiac	X	X	−22.6%	−25.0%	**2**	**12.0**
							3.0				−45.2%	−22.4%		
**24**	Ankeet Deepak Udani (2016)	Anesthesiology residents (28)	Randomized controlled trial design	Group 1: In‐person lectures with deliberate practice;Group 2: In‐person lectures only	O	1.5 hours	3.0	Ultrasound‐guided regional anaesthesia	X	X	X	Group 1: −13.16%; Group 2: −15.79%	**2**	**13.5**
**25**	James A. Town (2016)	Internal medicine residents (101)	Single‐group pretest and posttest design	In‐person lectures with case‐based discussion	O	2.5 hours	12.0	Vascular (deep venous thrombosis screening, volume status estimation)	X	−13.6%		X	**2**	**12.0**
**26**	Diana J. Kelm (2015)	PGY‐1 internal medicine residents (72)	Two group nonrandomized cohort design	In‐person lectures with case‐based discussion and clinical observation rotations	O	7 hours	6.0	Abdominal (ascites, kidney)	X	16.7%	X	X	**2**	**12.5**
								Thyroid		−17.9%				
								Lung (pleural fluid)		−7.1%				
								Vascular (IJV, IVC)		−8.8%				
**27**	Thomas Edrich (2014)	Anaesthesia residents, fellows, faculty (46)	Randomized controlled trial design	Group 1: In‐person lectures with video learning and simulator training; Group 2: In‐person lectures with video learning	O	1.3 hours	1.0	Cardiac	Group 1: −8.1%;Group 2: −3.6%			X	**2**	**13.5**
**28**	Scott M. Thomas (2013)	Postgraduate years 1–3 paediatric residents (26)	Single‐group pretest and posttest design	Video learning with small‐group practice	O	1.5 hours	3.0	Ultrasound‐Guided vascular access (CVC) ‐ Successful CVC insertion	X	X	X	−19.1%	**2**	**13.0**
**29**	Matthew Lyon (2012)	Prehospital care providers, registered nurses, critical care paramedics (8)	Single‐group pretest and posttest design	In‐person lectures with hands‐on practice on cadavar	O	0.4 hours	9.0	Lung (lung sliding sign)	X	X	3.2%		**2**	**10**
**30**	Oksana H. Baltarowich (2009)	Physicians (12)	Single‐group pretest and posttest design	In‐person lectures with learning by teaching, case reviews, informal conferences and clinical observation rotations	O	3 months	6.0	Abdomen	1.3%			X	**2**	**12.0**
								Obstetrics	7.1%					
								Gynaecology	0.8%					
**31**	Barry B. Goldberg (2001)	Physicians (112)	Single‐group pretest and posttest design	In‐person lectures with learning by teaching, case reviews, informal conferences and clinical observation rotations	O	3 months	6.0	Abdomen	8.0%			X	**2**	**11.0**
								Obstetrics	−1.9%					
								Gynaecology	6.2%					

### 3.2 Competence retention under the I‐AIM framework

Among all included studies, the Interpretation domain was the most frequently assessed component (*n* = 25); other domains—Acquisition (*n* = 15), Indication (*n* = 13) and Medical decision‐making (*n* = 9)—were less commonly evaluated as independent outcomes. Further analysis of competence retention revealed distinct patterns across different I‐AIM domains. The Acquisition domain demonstrated the most pronounced decline among all domains, with median percentage changes of −11.8% (IQR: −16.5% to −6.4%), reaching a plateau phase within the first 6 months' post‐intervention. Other domains exhibited varying retention patterns, but all showed negative trends. The Interpretation domain demonstrated an initial dramatic decline, with extreme deterioration reaching up to 45.7% at the 2‐month follow‐up^32^ and overall median percentage changes of −8.2% (IQR: −14.3% to −2.5%). Medical decision‐making and Indication domains showed comparatively modest declines, with median percentage changes of −3.7% (IQR: −7.2% to 0.1%) and −3.7% (IQR: −8.6% to −2.1%), respectively (Appendix S1).

### 3.3 Performance impact of educational duration

We analysed and classified the educational duration of all studies using a 4‐h threshold, as the majority (81.4%) of curricula lasted <4 h. Overall, 43 training courses were categorized into short‐duration (≤4 h, *n* = 35) and long‐duration (>4 h, *n* = 8) groups (Appendix S2). Participants demonstrated significantly greater competency decline with short courses, showing a median percentage change of −11.8% (IQR: −16.9% to −4.4%), compared with long courses at −2.6% (IQR: −6.8% to 1.3%). The evaluation intervals also revealed a marked difference, with short courses assessed at a median of 2 (IQR: 1 to 3) months; long courses were evaluated at a median of 6 months.

The differential patterns of competency decline stratified by educational duration were consistently observed across all domain‐specific skills within the I‐AIM framework. The Acquisition domain exhibited the most pronounced deterioration, with a median percentage change of −12.5% (IQR: −17.3% to −10.3%) for short courses compared with −5.8% (IQR: −9.1% to −2.6%) for long courses. Similar trends were observed across other domains: Interpretation (−10.2% vs − 2.3%; IQR: −17.2% to −3.8% vs − 8.4% to 1.2%), Medical decision‐making (−8.1% vs − 1.9%; IQR: −15.2% to −4.1% vs − 3.7% to 3.8%) and Indication (−6.6% vs − 3.1%; IQR: −12.8% to −3.8% vs − 4.6% to 2.5%).

### 3.4 Performance impact of educational methodologies

#### 3.4.1 Integrating hands‐on practice

While hands‐on practice was a universal component in Acquisition skill training, its benefits extended well beyond this domain. The integration of practical training components led to significant performance improvements across other core domains—including Indication knowledge (median: −3.7% vs − 8.9%; IQR: −8.3% to −1.2% vs − 11.1% to −6.7%) and skills for Medical decision‐making (median: −3.7% vs − 8.9%; IQR: −4.8% to 0.9% vs − 11.1% to −6.7%)—when compared to curricula without practical training sessions. Conversely, the difference in Interpretation abilities was less pronounced (median: −8.5% vs − 8.0%; IQR: −17.1% to −2.5% vs − 13.5% to −3.8%) (Appendix S3). Additionally, participants exhibited superior retention outcomes in the Acquisition domain when advanced methodologies, such as simulators, three‐dimensional models, or cadaver‐based practice, were incorporated, compared to traditional hands‐on approaches (Appendix S4). Thampi et al (2020) demonstrated that simulator‐based training combined with three‐dimensional model‐assisted lectures resulted in a minimal decline of 0.5% in transesophageal echocardiography (TEE) acquisition skills after 1 month.^8^ Kule et al (2022) documented a 36.5–68% improvement in the success rate of ultrasound‐guided peripheral intravenous catheter placement over 6 months among 48 medical students following simulation‐based mastery learning with four to eight additional training sessions and real human assessments.^21^ A larger‐scale study by Schott et al (2021) involving 127 practicing physicians revealed excellent retention in ultrasound‐guided intravenous catheter placement skills using simulation task trainers, with minimal skill decay of only 2.6% over 8 months.^33^ Furthermore, Lyon et al (2012) highlighted the effectiveness of cadaver‐based training, reporting a 3.2% improvement in lung sliding sign identification skills after 9 months among prehospital care providers.^20^

However, several studies yielded contradictory findings. Zhao et al (2021) found that simulator‐based training for TEE interpretations led to greater skill decay (−17.3%) compared to video‐based instruction (−8.2%) at the 1‐month follow‐up.^34^ Similarly, Ochoa et al (2019) reported that the integration of three‐dimensional heart models in lectures resulted in more pronounced skill deterioration (−35.6%) than traditional lecture methods (−24.3%).^35^ Thomas et al (2013) also documented a skill decline (−19.1%) in ultrasound‐guided central venous catheter placement among 26 paediatric residents over a 3‐month period using high‐fidelity paediatric vascular access simulators.^36^ Furthermore, Edrich et al (2014) demonstrated that supplementary simulator training did not enhance cardiac ultrasound skill retention, showing a greater decline (−8.1%) compared with standard training (−3.6%) at the 1‐month assessment.^37^

#### 3.4.2 Integrating clinical context

Training programs, including the studies by Baltarowich et al (2009), Goldberg et al (2001) and Kelm et al (2015), which incorporated case reviews, case‐based discussions, or clinical rotations—despite requiring extended durations (ranging from 7 h to 3 months)—demonstrated enhanced outcomes across multiple competency domains.^10^, ^38^, ^39^ These integrated approaches proved more effective in Interpretation (median: 3.8% vs − 9.1%; IQR: 0.9% to 6.9% vs − 16.6% to −3.5%), Medical decision‐making (median: 3.8% vs − 4.1%; IQR: 0.9% to 6.9% vs − 10.8% to −3.7%) and Indication (median: 1.1% vs − 4.1%; IQR: −5.8% to 6.9% vs − 10.3% to −3.7%) domains compared to other reviewed training programs (Appendix S5). In contrast, Town et al (2016) reported a 13.6% decline in the Interpretation and Indication domains within a 12‐month follow‐up after a condensed 2.5‐h vascular PoCUS curriculum with case‐based discussions.^40^

## 4 DISCUSSION

Over recent decades, PoCUS has evolved into a fundamental skill for healthcare providers and has become indispensable across multiple medical specialties.^8^, ^12^, ^20^, ^41^ This systematic review reveals distinctive patterns in PoCUS competency retention, demonstrating that skill deterioration remains a significant educational challenge. Utilizing the I‐AIM framework, we found that the Acquisition domain, which involves mainly the psychomotor skills necessary for optimal patient positioning and precise probe manipulation, showed the most pronounced decline (−11.8%), followed by Interpretation abilities (−8.2%). The more rapid deterioration in the Acquisition domain, compared to cognitive knowledge retention, may be attributed to inherent differences between physical and cognitive tasks.^42^ Cognitive domains, such as Indication, Interpretation and Medical decision‐making, may be better retained through mental rehearsal and contextual reinforcement, both of which have been shown to mitigate skill decay. In contrast, physical tasks such as image acquisition require continual hands‐on practice to maintain proficiency.^43^ This phenomenon aligns with previous findings in various medical procedures, including adult ALS,^22^ basic life support,^44^, ^45^, ^46^, ^47^ neonatal resuscitation programs,^48^ and emergency obstetric care.^49^

A few studies documented sustained or enhanced competency levels over time, indicating knowledge or skill enhancement rather than the anticipated deterioration. Multiple investigators, including Baltarowich et al and Goldberg et al, attributed this phenomenon to two potential factors: first, their study population comprised more experienced physicians; second, participants were provided with comprehensive educational materials post‐training and were encouraged to serve as program directors. This “Learning as Teachers” approach may have contributed to sustained skill retention and continued improvement through active engagement in educational roles.^38^, ^39^

Our analysis revealed that 81.4% of training courses (*n* = 35) implemented short courses of ≤4 h, presumably owing to resource constraints, clinical workload demands, or other contextual limitations. This model showed a substantial overall competency decline, with a median percentage change of −11.8% (IQR: −16.9% to −4.4%), most notably in Acquisition skills (−12.5%, IQR: −17.3% to −10.3%). Contrastingly, the remaining 18.6% of training courses (*n* = 8) utilized long courses (>4 h) and demonstrated superior retention outcomes (−2.6%, IQR: −6.8% to 1.3%) over extended follow‐up periods of 6 months. These findings emphasize the need for just‐in‐time refresher training for novice practitioners, particularly with abbreviated curricula (≤4 h), rather than solely relying on one‐time foundational instruction. A longitudinal training curriculum incorporating a “bolus and drip” approach emerges as a promising pedagogical strategy, effectively integrating structured didactic materials (“bolus”) with practical scanning sessions (“drip”) to facilitate long‐term competency.^50^ This training paradigm aligns with established practices in ALS training, where brief, frequent educational interventions have demonstrated enhanced efficacy in both cardiopulmonary resuscitation performance and skill retention.^51^, ^52^ Nevertheless, comparative effectiveness studies between the “bolus and drip” approach and traditional extended‐duration training protocols warrant further investigation to establish definitive evidence‐based recommendations.

Hands‐on practice emerged as a fundamental component in PoCUS education, enhancing not only Acquisition skills but also strengthening performance across multiple competency domains, particularly Indication and Medical decision‐making. However, its impact on Interpretation abilities was less pronounced, suggesting the need for complementary strategies to further support this specific skill. Previous research has substantiated the irreplaceable role of hands‐on practical training in developing healthcare professionals' confidence, knowledge base and proficiency in medical image acquisition and interpretation.^23^, ^53^, ^54^, ^55^ Furthermore, the integration of high‐fidelity simulation tools, including simulators, three‐dimensional models and cadaver‐based practice, appears to facilitate sustained acquisition ability, consistent with literature demonstrating enhanced participant engagement and learning outcomes.^56^, ^57^

The distinctive characteristics of PoCUS—real‐time assessment capability, portability and absence of radiation exposure—have transformed it beyond a conventional ultrasound examination into “the new stethoscope” for contemporary healthcare providers. In clinical practice, the I‐AIM framework guides practitioners through a systematic approach: first evaluating clinical benefits and determining appropriate diagnostic imaging tools through careful history‐taking and physical examination (**Indication**); then performing targeted sonographic examinations to acquire optimal images (**Acquisition**); simultaneously conducting systematic interpretation of sonographic images to diagnose pathological lesions, supplementing traditional physical examination methods (**Interpretation**); and finally, synthesizing all clinical information for appropriate medical management (**Medical decision‐making**).^24^ This intrinsic connection between PoCUS and clinical practice emphasizes the critical importance of early **clinical context integration** during training. Our findings demonstrate that incorporating case reviews, clinical discussions and bedside rotations significantly enhances participants' PoCUS competence across I‐AIM domains, including Indication, Interpretation and Medical decision‐making. Such integration effectively bridges theoretical knowledge with real‐world clinical scenarios, providing cognitive scaffolding that enhances both learner motivation and educational outcomes.^58^ However, this comprehensive training methodology necessitates extended durations, ranging from 7 h to 3 months, indicating increased resource requirements.

These findings highlight the importance of designing PoCUS training programs that comprehensively address all domains of the I‐AIM framework, rather than concentrating primarily on image interpretation as seen in many existing studies. To promote long‐term retention of PoCUS competence—particularly in the Acquisition domain, where psychomotor skills are most prone to decay—we recommend the implementation of integrated PoCUS curricula that incorporate multi‐modal educational strategies, supplemented by scheduled refresher training, emphasizing longitudinal and structured didactic content for all I‐AIM domains and supervised scanning sessions for image acquisition. The integration of simulation‐based practice utilizing high‐fidelity simulators, coupled with authentic clinical scenarios, appears to optimize learner competency across the I‐AIM framework domains. Moreover, future studies should employ rigorous evaluation methods, particularly those aligned with Kirkpatrick's Levels 3 (behavioural change) and 4 (clinical outcomes), to assess the transfer of learning to clinical practice and its impact on patient care.

Several methodological limitations warrant careful consideration in interpreting our findings. The predominance of single‐group pretest‐posttest designs (54.8%) and the scarcity of studies achieving Kirkpatrick's Level ≥3 evaluation outcomes represent significant methodological constraints. The substantial heterogeneity across studies—encompassing variations in educational interventions, measurement intervals, non‐validated assessment tools and diverse scoring systems—impedes meaningful cross‐study comparisons and precludes comprehensive meta‐analysis. Although we attempted to standardize results through mean percentage change calculations (Δ%), other methodological variations may introduce potential bias. The study populations, predominantly composed of medical students (38.6%), potentially limit the generalizability of findings to other healthcare professional populations. Furthermore, the relatively brief follow‐up periods—median 2 (IQR: 1–3) months for short courses and 6 months for long courses, rarely exceeding 12 months—limit our understanding of long‐term skill retention patterns. The disproportionate emphasis on Interpretation and Acquisition domains in most studies hampers comprehensive evaluation of structured PoCUS competence across all framework components.

These limitations highlight critical directions for future research. Although the I‐AIM framework has been widely adopted in clinical practice across various settings, few studies have rigorously evaluated its validity and reliability.^26^ Future research should focus on developing and validating standardized assessment tools for each I‐AIM domain, particularly the less‐explored areas of Acquisition, Indication and Medical decision‐making. Studies should also examine long‐term PoCUS competency retention across diverse healthcare provider populations and incorporate extended follow‐up periods. A more robust evaluation approach is essential to deepen our understanding of the mechanisms by which different domains contribute to the sustained competence of PoCUS practitioners.

In summary, PoCUS competency retention presents a significant educational challenge, with the I‐AIM framework providing a structured assessment approach. Acquisition and Interpretation skills show the most rapid decay, particularly in short‐course training programs (≤4 h). Our findings support implementing comprehensive PoCUS training programs that integrate multi‐strategy curricula with both high‐fidelity simulation practice (using simulators, three‐dimensional models and cadaver‐based training) and clinical context through case reviews, discussions and bedside rotations to optimize competency retention across I‐AIM framework domains. These insights provide valuable guidance for medical educators in developing effective PoCUS training programs and informing future educational strategies.

## AUTHOR CONTRIBUTIONS

LWW, CHL, YLC, HLC and CWY conceptualized and designed the study. LWW and CHL conducted the systematic literature search and screening, performed data extraction and curated the data. Formal analysis was conducted by LWW, CHL, WYL, WCC and MHMM. Funding acquisition was led by LWW and CWY. CHL and WYL provided resources and software support. Validation was performed by WCC and MHMM, while visualization was prepared by YLC and HLC. LWW drafted the initial manuscript, and all authors reviewed and revised the manuscript critically for important intellectual content. YLC, HLC and CWY supervised the study and provided methodological guidance. All authors approved the final manuscript and agreed to be accountable for all aspects of the work.

## CONFLICT OF INTEREST STATEMENT

The authors have no conflicts of interest to declare that are relevant to the content of this article.

## ETHICS STATEMENT

This study is a systematic review of previously published literature. No human participants or animals were involved and no new data were collected. Therefore, ethical approval or informed consent was not required.

## Supporting information


**Appendix S1.** Scatter plot of I‐AIM domains.

**Appendix S2.** Mean percentage changes in educational outcomes stratified by course duration.

**Appendix S3.** Mean percentage changes in educational outcomes comparing hands‐on practice integration.

**Appendix S4.** Mean percentage changes in Acquisition performance were evaluated following educational interventions, comparing simulation‐based methods with traditional practice methods.

**Appendix S5.** Mean percentage changes in educational outcomes comparing clinical integration methods.

## Data Availability

The data that support the findings of this study are available from the corresponding author upon reasonable request.
