# Supplementary material for: Is ultrasound training sustainable? A systematic review of competency retention in healthcare trainees
Source: Med Educ. 2025 Jun 16;59(12):1290–305. doi: 10.1111/medu.15751 (PMC12686767; doi:10.1111/medu.15751)
Supplement: Supplementary file 1 — Appendix S1. Scatter plot of I‐AIM domains. [file MEDU-59-1290-s002.pdf]

Appendix 1. Scatter plot of I-AIM domains.

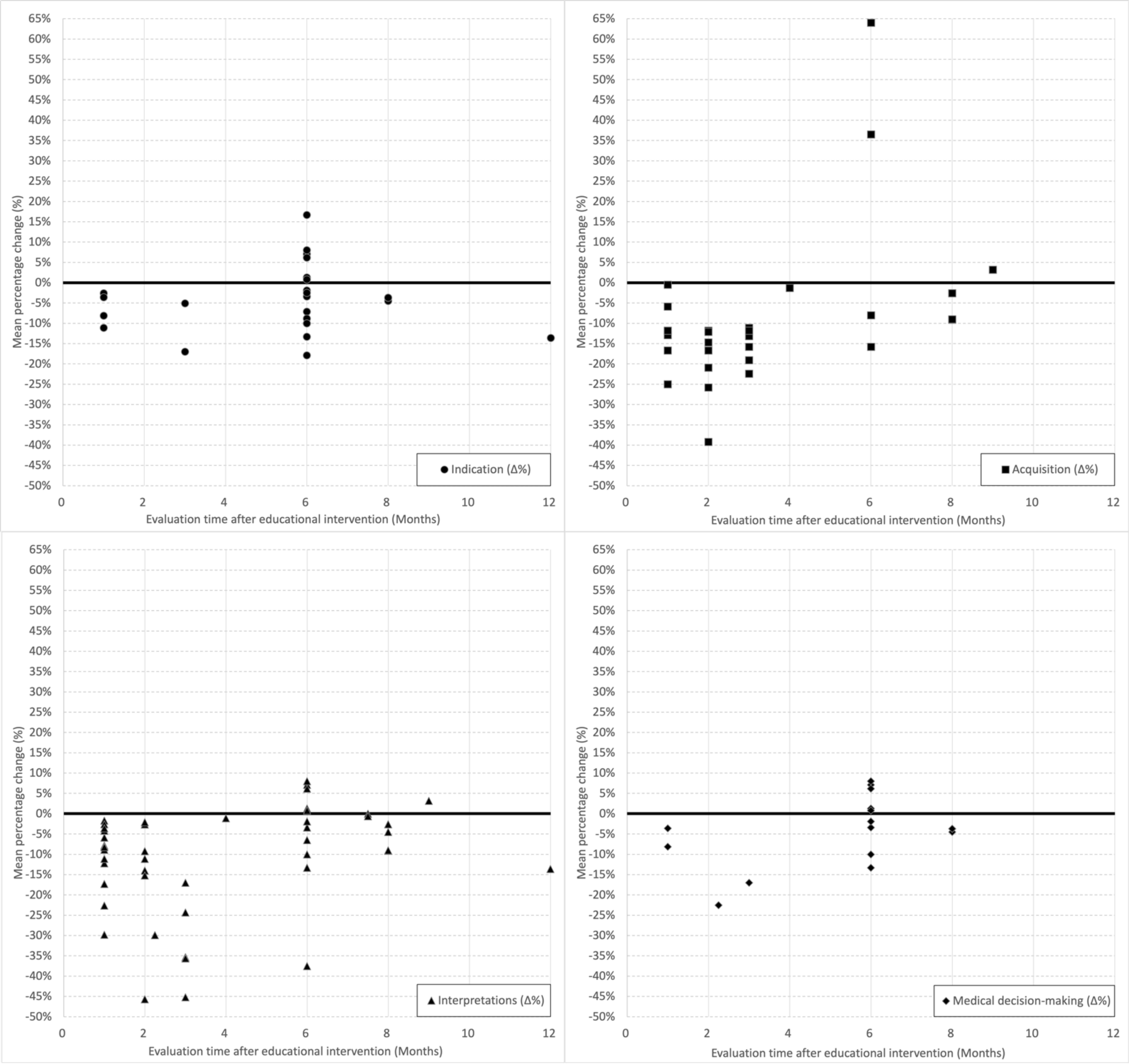

Mean percentage changes in four domains (Indication, Acquisition, Interpretations, and Medical decision-making) after educational intervention. The scatter plot displays evaluation time points (months) on the x-axis and mean percentage changes (%) on the y-axis. Each marker represents an individual measurement, with circles indicating Indication, squares indicating Acquisition, triangles indicating Interpretations, and diamonds indicating Medical decision-making. The horizontal black line represents the baseline (0% change).
