# Supplementary material for: Is ultrasound training sustainable? A systematic review of competency retention in healthcare trainees
Source: Med Educ. 2025 Jun 16;59(12):1290–305. doi: 10.1111/medu.15751 (PMC12686767; doi:10.1111/medu.15751)
Supplement: Supplementary file 2 — Appendix S2. Mean percentage changes in educational outcomes stratified by course duration. [file MEDU-59-1290-s001.pdf]

Appendix 2. Mean percentage changes in educational outcomes stratified by course duration

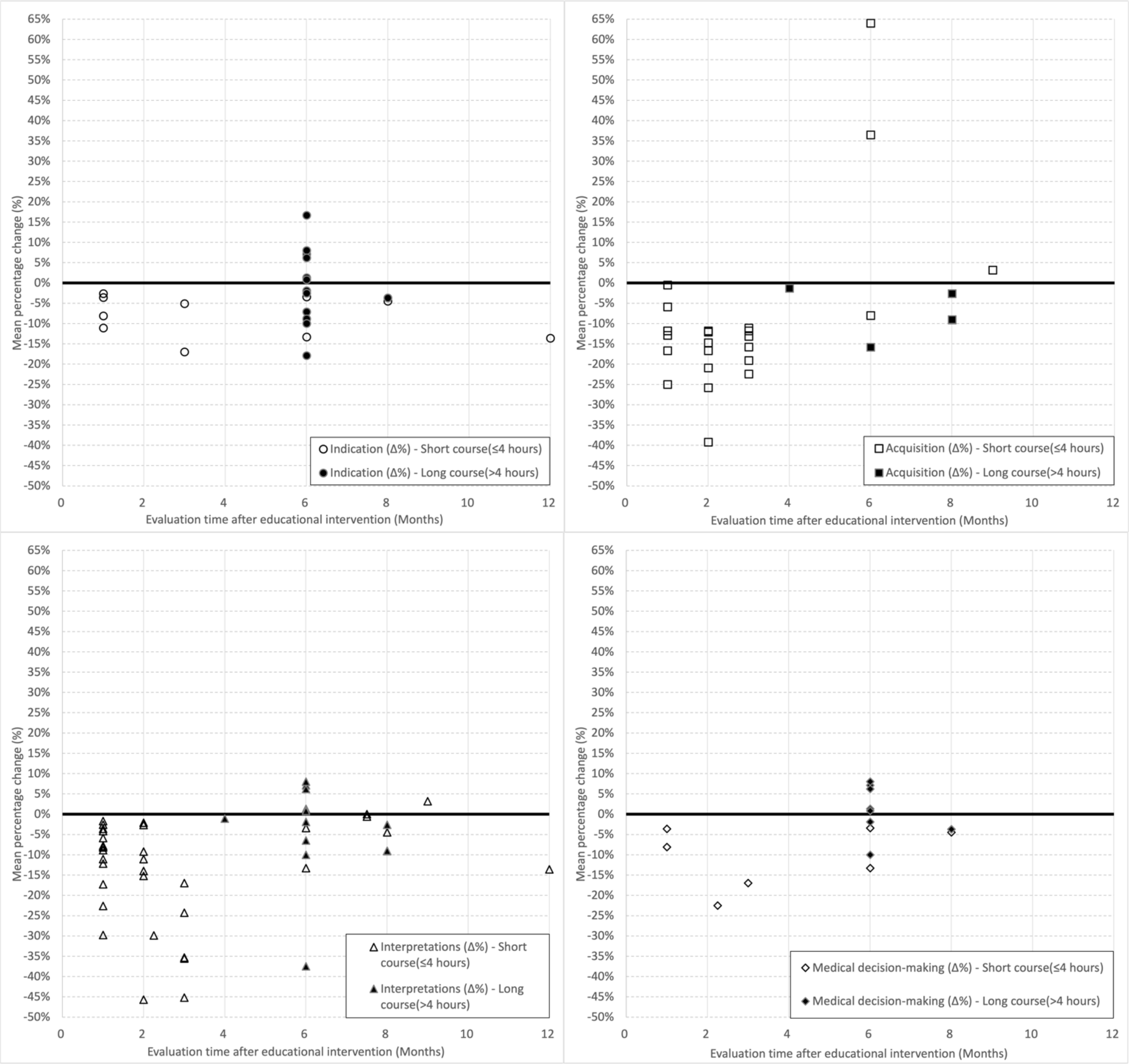

Mean percentage changes in four domains (Indication, Acquisition, Interpretations, and Medical decision-making) after educational intervention, comparing short course (≤4 hours) and long course (>4 hours) effects. Different markers indicate the domains and course duration: hollow circles for Indication (short course), filled circles for Indication (long course); hollow squares for Acquisition (short course), filled squares for Acquisition (long course); hollow triangles for Interpretations (short course), filled triangles for Interpretations (long course); and hollow diamonds for Medical decision-making (short course), filled diamonds for Medical decision-making (long course). The horizontal black line represents the baseline (0% change).
