# Supplementary material for: Is ultrasound training sustainable? A systematic review of competency retention in healthcare trainees
Source: Med Educ. 2025 Jun 16;59(12):1290–305. doi: 10.1111/medu.15751 (PMC12686767; doi:10.1111/medu.15751)
Supplement: Supplementary file 4 — Appendix S4. Mean percentage changes in Acquisition performance were evaluated following educational interventions, comparing simulation‐based methods with traditional practice methods. [file MEDU-59-1290-s005.pdf]

Appendix 4. Mean percentage changes in Acquisition performance were evaluated following educational interventions, comparing simulation-based methods with traditional practice methods.

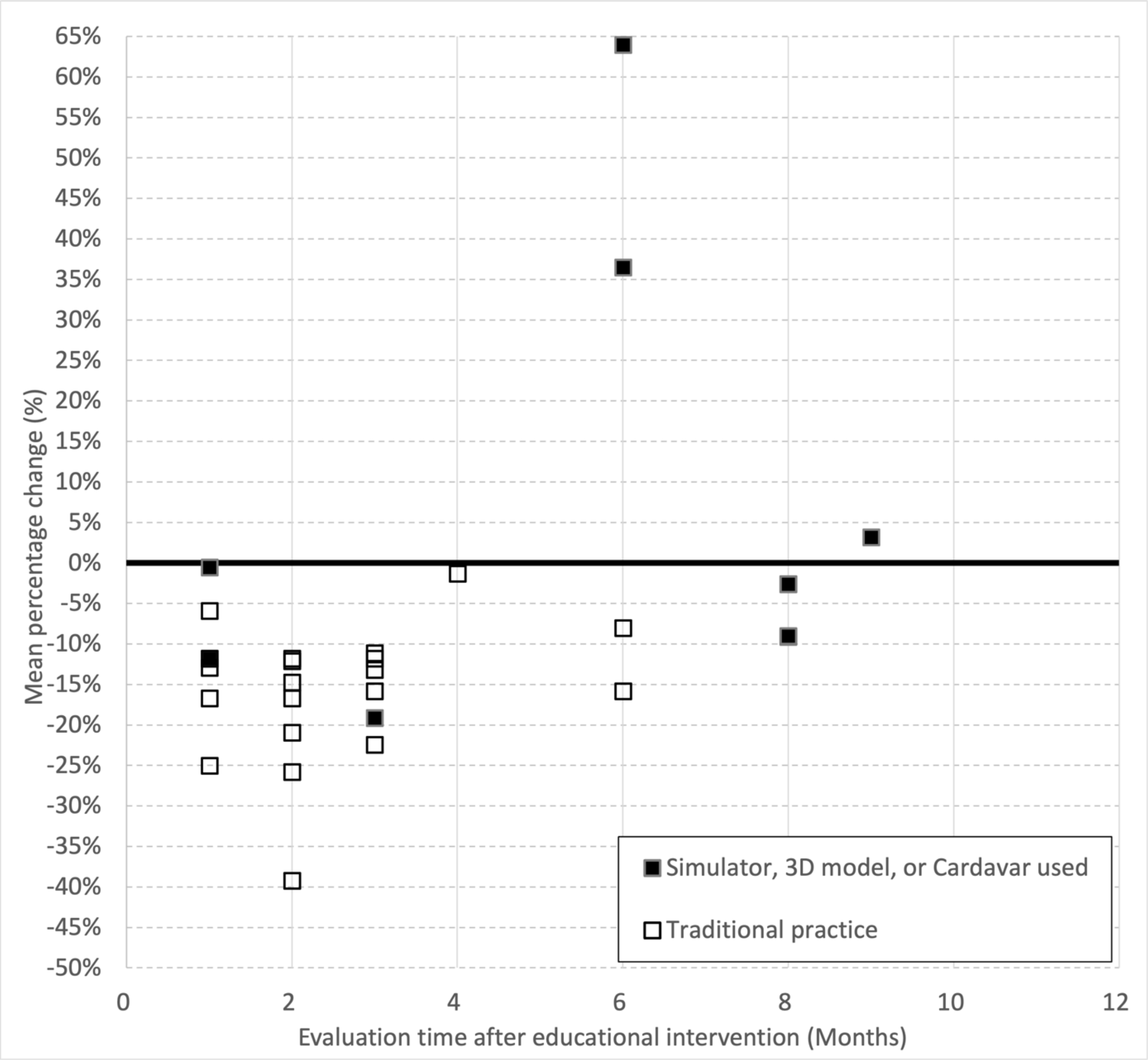

Data points are depicted as black squares for simulation-based practice groups and white squares for traditional practice groups. The x-axis represents the evaluation time (in months) following the educational intervention, while the y-axis illustrates the mean percentage change in performance (%). The horizontal black line represents the baseline (0% change).
