# Supplementary material for: Is ultrasound training sustainable? A systematic review of competency retention in healthcare trainees
Source: Med Educ. 2025 Jun 16;59(12):1290–305. doi: 10.1111/medu.15751 (PMC12686767; doi:10.1111/medu.15751)

Appendix 5. Mean percentage changes in educational outcomes comparing clinical integration methods

Mean percentage changes in three domains (Indication, Interpretations, and Medical decision-making) after educational intervention, comparing groups with and without clinical integration. Different markers indicate integration status: filled circles for groups with clinical integration, and hollow circles for groups without clinical integration. The data is presented in three separate panels by domain: the left panel for Indication, the middle panel for Interpretations, and the right panel for Medical decision-making. The horizontal black line represents the baseline (0% change).

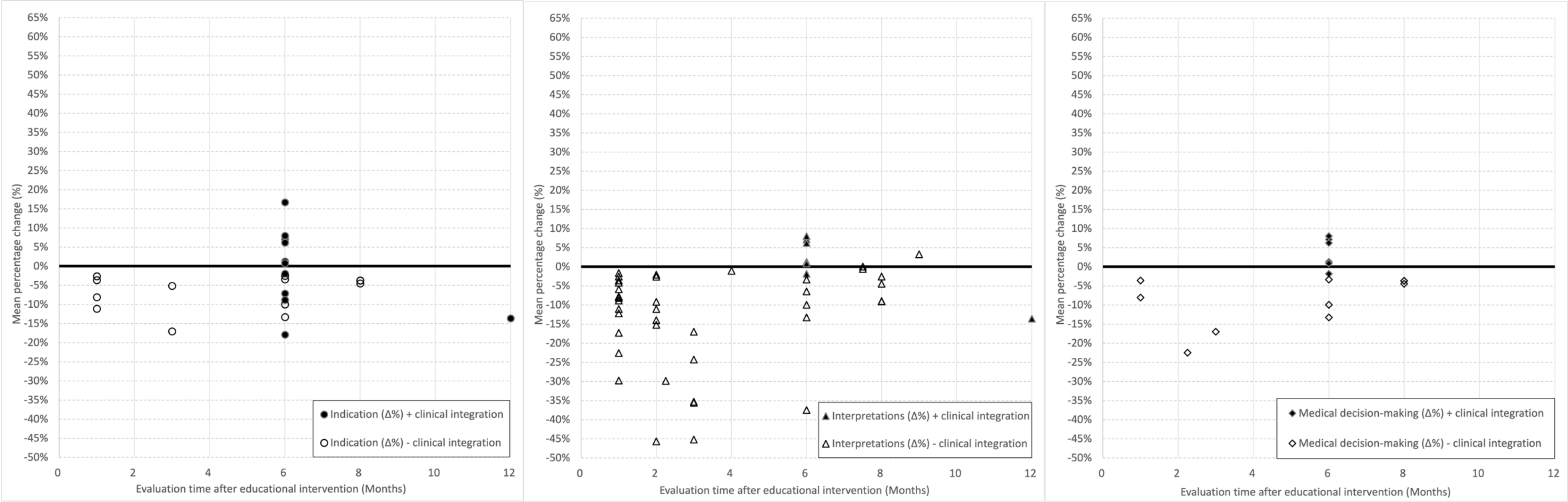

Supplement: Supplementary file 5 — Appendix S5. Mean percentage changes in educational outcomes comparing clinical integration methods. [file MEDU-59-1290-s003.pdf]
